# Perspective: Challenges Presented for Regeneration of Heterogeneous Musculoskeletal Tissues that Normally Develop in Unique Biomechanical Environments

**DOI:** 10.3389/fbioe.2021.760273

**Published:** 2021-09-28

**Authors:** David A. Hart, Norimasa Nakamura, Nigel G. Shrive

**Affiliations:** ^1^ Department of Surgery, Faculty of Kinesiology, University of Calgary, Calgary, AB, Canada; ^2^ Bone and Joint Health Strategic Clinical Network, Alberta Health Services, Edmonton, AB, Canada; ^3^ McCaig Institute for Bone & Joint Health, University of Calgary, Calgary, AB, Canada; ^4^ Institute for Medical Science in Sport, Osaka Health Science University, Osaka, Japan; ^5^ Biomedical Engineering Graduate Program, Department of Civil Engineering, University of Calgary, Calgary, AB, Canada

**Keywords:** musculoskeletal regeneration, tissue Engineering, biomechanics, tissue heterogeneity, complex tissue organization

## Abstract

**Perspective:** Musculoskeletal (MSK) tissues such as articular cartilage, menisci, tendons, and ligaments are often injured throughout life as a consequence of accidents. Joints can also become compromised due to the presence of inflammatory diseases such as rheumatoid arthritis. Thus, there is a need to develop regenerative approaches to address such injuries to heterogeneous tissues and ones that occur in heterogeneous environments. Such injuries can compromise both the biomechanical integrity and functional capability of these tissues. Thus, there are several challenges to overcome in order to enhance success of efforts to repair and regenerate damaged MSK tissues.

**Challenges:** 1. MSK tissues arise during development in very different biological and biomechanical environments. These early tissues serve as a template to address the biomechanical requirements evolving during growth and maturation towards skeletal maturity. Many of these tissues are heterogeneous and have transition points in their matrix. The heterogeneity of environments thus presents a challenge to replicate with regard to both the cells and the ECM. 2. Growth and maturation of musculoskeletal tissues occurs in the presence of anabolic mediators such as growth hormone and the IGF-1 family of proteins which decline with age and are low when there is a greater need for the repair and regeneration of injured or damaged tissues with advancing age. Thus, there is the challenge of re-creating an anabolic environment to enhance incorporation of implanted constructs. 3. The environments associated with injury or chronic degeneration of tissues are often catabolic or inflammatory. Thus, there is the challenge of creating a more favorable in vivo environment to facilitate the successful implantation of in vitro engineered constructs to regenerate damaged tissues.

**Conclusions:** The goal of regenerating MSK tissues has to be to meet not only the biological requirements (components and structure) but also the heterogeneity of function (biomechanics) in vivo. Furthermore, for many of these tissues, the regenerative approach has to overcome the site of injury being influenced by catabolism/inflammation. Attempts to date using both endogenous cells, exogenous cells and scaffolds of various types have been limited in achieving long term outcomes, but progress is being made.

## Introduction

The repair or reconstitution of injured or damaged musculoskeletal (MSK) connective tissues has long been a goal of orthopedic surgeons, plastic surgeons, other health care providers and researchers. Many acutely injured MSK tissues heal naturally with formation of scar tissue which in many cases is severely compromised with regard to function, or they heal poorly due to very limited vascularity or innervation. Alternatively, surgeons have explored the use of transplanting allogeneic normal tissues in place of the injured or damaged ones. Such approaches to reconstitute torn anterior cruciate ligaments (ACL), damaged menisci of the knee, or articular cartilage has met with some success over the relatively short term, but even replacement of the ACL with an autologous piece of a tendon [e.g., middle third of the patellar tendon] leads to the stretching out (irreversible creep) of the tendon over time with loss of function.

Thus, there remains the challenge of developing new approaches to tissue engineer replacement connective tissues using autologous somatic cells + scaffolds of defined structure and content, autologous or allogeneic mesenchymal stem cells ± defined scaffolds, or other variations of cells± scaffolds or relevant extracellular matrix components. While such approaches have led to some successes, the field is still evolving and success, depending on the definition, is variable. In part, this variable success relates to whether one aspires to restoration of biological and/or mechanical long-term function. Of course, the ideal would be regeneration of the tissues injured via accidents or disease, but this challenge still persists, in part due to incomplete appreciation of the complexities of these tissues in different environments, and some confusion as to how to recapitulate development and maturation in a skeletally mature adult who may be normal or abnormal due to chronic disease. Finally, progress in the repair and regeneration of injured or damaged human connective tissues may be compromised by the dependence on small preclinical models that rely more on biological outcomes than ability to assess the integrity of the biomechanical function of the engineered replacement tissues.

### Connective Tissue Heterogeneity

Connective tissues of the mammalian MSK system have evolved to allow for functioning in the one gravity (1 g) environment of Earth in a coordinated and integrated manner. Humans further adapted to walk upright with an accompanying ability to be highly mobile.

While the initial biological mechanisms that allowed individual cells to adapt to the 1 g environment of Earth evolved billions of years ago, they have been incorporated into increasingly complex organisms via multicellular organisms, various sea creatures, dinosaurs, birds, early mammals, primates and various iterations of humans. Thus, in humans, tissues of the MSK system range from hard tissue such as bone, to soft tissues such as muscles, tendons, ligaments, intervertebral discs, articular cartilage, and menisci. Not only do some of these tissues exhibit heterogeneity within a specific tissue functioning in its unique environment, but for the tissues of a joint such as the knee, the individual tissues must also work together for optimal functioning (i.e., the Joint as an Organ System; Radin et al., 1991; Frank et al., 2004; Loesser et al., 2012).

For tissues such as tendon, which connect muscles to bones, the tissues consist of a myotendinous junction, a mid-substance, and an insertion into bone. With multiple transition points, there is heterogeneity in the content of the tissue and its organization, heterogeneity that can change with age (Fallon et al., 2002; Kostrominova and Brooks, 2013; Thornton et al., 2015; Huisman et al., 2014). Furthermore, tendons operating in different mechanical environments, such as the Achilles tendon and the Supraspinatus (SS) tendon, appear to exhibit different properties (Thornton et al., 2010; Fallon et al., 2002). Other tendons that traverse around a boney prominence have a different structure at the site of such compression (Vogel and Koob, 1989; Vogel 2003, 2004; Vogel and Peters, 2005). That is, at sites of compression, the extracellular matrix becomes more cartilage-like with expression of type II collagen and an organization appropriate for addressing compressive rather than tensile loading. In addition, if the tissue is taken off the boney prominence, it undergoes a reversible transformation of its matrix. Interestingly, injuries to the SS tendon occur mainly at the transition of the tendon into bone, or the enthesis, which is a very unique transition tissue (Sevick et al., 2018, 2021).

Tendons are not unique with regard to this heterogeneity and variation in functioning in different environments. Certainly bone can adapt to its mechanical environment (discussed in Frost, 1996, 2004), muscles differ in their fibre types and metabolism via mitochondria content, and skin can vary depending on its location and use. Thus, connective tissues which may look fairly homogenous are in fact quite heterogeneous at multiple levels, Furthermore, many of these tissues also vary in response to transitions such as puberty, pregnancy, and menopause and are thus influenced by sex hormones (discussed in Rollick et al., 2018).

The ligaments of the knee are also heterogeneous. The medial collateral ligament (MCL) is a passive, extra-articular structure which stabilizes the knee and typically operates in the toe region of its load displacement curve [discussed in Bray et al., 2005]. In contrast, the anterior cruciate ligament (ACL) operates in the intra-articular space and experiences higher load levels than the MCL (discussed in Wan et al., 2014; Mallett and Arruda, 2017). Loading also depends on the angle of the knee. Similar variation in normal loading occurs in tendons in different environments as well. For both ligaments and tendons, their normal loading can range from kPa to mPa, while those for articular cartilage is in the MPa range. Certainly, the complex structure of the ACL, a ligament which is often injured, is also difficult to replicate.

Another example of a heterogeneous tissue is a meniscus of the knee (Andrews et al., 2014; Andrews et al., 2017; Brzezinski et al., 2017; Arnoczky et al., 2010; Murphy et al., 2019, Rattner et al., 2011). In addition, age-related changes in menisci may further complicate the healing potential and may also influence the potential for success when tissue engineering approaches are attempted (Di Giancamillo et al., 2017).

Thus, a first challenge for those interested in regeneration of injured connective tissues is related to the tissue heterogeneity and the need to understand the relationships between that heterogeneity and function. For example, no two ligaments are exactly the same–they are each structured to do their specific job in unique biomechanical environments. Thus, such heterogeneity is likely tissue-specific, which could pose unique issues regarding the approaches taken to develop replacement tissues and regain function in different environments (Table 1).

**TABLE 1:** Challenges for tissue engineering success for repair/regeneration of musculoskeletal tissues.

Challenge	Potential solutions
1. Tissue Heterogeneitya Biologic	a) General template that allows for adaptation to *in vivo* biologic and mechanical cues
	b) Use of decellularized natural matrix + Progenitor cells capable of differentiation
	c) Facilitate integration to residual normal tissue-fibronectin/RGD sequences?
	d) Derivatize matrix with developmentally regulated anabolic mediators (i.e., IGF-1 family + IGF-1 binding proteins)
Biomechanical Environment	a) Apply relevant loading *in vitro* during development that corresponds to *in vivo* requirements
	b) Progressive *in vitro* loading increases to ensure survival post-implantation and enhance cellular response pattern
2. Complexity of ECM-Cell Organizationb	a) Use of decellularized natural matrix (ECM) +/- synthetic matrix elements that can be reconstituted with Progenitor or Normal cells
	b) 3D printing of natural ECM mimetic with immune privileged Progenitor cells
	c) Supplementation with a stable supply of developmentally regulated anabolic signals (i.e., slow release from a matrix store)
3. Control an Adverse *in vivo* Environment (i.e., Inflammation)	a) Exposure of *in vivo* environment to appropriate drugs to control the inflammatory environment (i.e., glucocorticoids) at the time of implantation
	b) Derivatize the matrix with slow release immunomodulatory cytokines and/or drugs
	to sustain an *in vivo* anti-inflammatory environment
	c) Inject autologous Platelet-rich Plasma at the time of implantation and as needed to both exert an immunomodulatory effect as well as provide anabolic signals
	d) Expose the implantation site to immunomodulatory exosomes from Progenitor Cells to suppress a catabolic environment and provide anabolic signals

a Such as a tendon with a bony enthesis, mid-substance and myotendinous junction to muscle; or a meniscus of the knee with a cartilaginous inner section and an outer more ligamentous section.

b Such as a knee meniscus with a complex array of tie fibers and associated fibers (Andrews et al., 2014; Andrews et al., 2017) and colonized with cells in different configurations (Hellio Le Graverand et al., 2001).

### Origin of Connective Tissue Heterogeneity and Organization During Development and Maturation

During fetal development the cellularity and organizational template for the connective tissues of the MSK system are laid down. For humans, after the articulating joints have developed and movement of the joints is achieved, the loading of the joint tissues (i.e., of the knee-ligaments, menisci, capsule, cartilage, bone, etc) in the absence of ground reaction forces still leads to further maturation in utero. At birth, there is considerable variation in the level of maturation of the tissues of different mammals (e.g., humans do not learn to walk for months while a young zebra or wildebeest is up and running in minutes).

When a rabbit is born, tissues such as the medial collateral ligament (MCL) are “cell rich and matrix poor” and during maturation *via* loading and growth, the MCL rapidly becomes “cell limited and matrix rich” (Meller et al., 2009; Ionescu et al., 2011). Interestingly, the DNA content of the MCL shortly after birth is very similar to that of the MCL of the skeletally mature adult so the tissue grows in a patterned manner coordinated with its loading environment: matrix is laid down between cells building on the original template. As the cells grow apart due to ECM deposition, they retain a cellular network via gap junctions (Lo et al., 2002): thus even if they look well separated on an H&E section of the tissue, they can still be in communication. Depriving the MCL of loading during growth and maturation leads to a cessation of growth (reviewed in Hart et al., 2002), so loading is necessary for a tissue to respond to whatever anabolic signals are present to enhance growth.

In menisci, cells also add to the complex template of matrix after birth, but the distribution of vascularization and innervation is also altered. At birth, the complete meniscus is innervated and vascularized, but as growth continues with loading, the inner aspect of the menisci gradually become avascular and aneural (discussed in Di Giancamillo et al., 2014). This inner aspect of the tissue is subjected to high compressive stresses between the femoral and tibial articular cartilage and low tensile circumferential stress, while the outer aspects are subjected to high circumferential tensile loading (“hoop stresses”). Thus, the tissue structure needs to “transition” in form between the inner and outer aspects to carry the different dominant stresses in those regions. The connectivity between cells is also different in these different areas of the menisci (Hellio Le Graverand et al., 2001). The loss of vascularity in the inner aspect of the menisci is believed to contribute to their lack of healing when injured as an adult (Di Giancamillio et al., 2017).

Other connective tissues of the MSK system also appear to follow a similar pattern for growth and maturation. The consequence is tissues with unique ECM content and cellularity dependent on the unique loading environments in which they are expected to function, and within the “physiologic window” of loading that is associated with homeostasis in that particular environment. For all connective tissues, except for articular cartilage which is aneural and avascular, the cells in the tissues can be endogenous “fibroblast-types”, microvascular endothelial cells, pericytes that can be either called mesenchymal stem cells or medicinal signaling cells (Caplan, 2017, 2019), all working together to allow for functioning in the 1 g environment of Earth.

After skeletal maturation, some connective tissues can continue to adapt and change over time. This may relate to epigenetic changes based on experiences of specific tissues (Peffers et al., 2016; Naue et al., 2018), or in the case of some tendons, loss of expression of the lubricant PRG4 can affect the gliding of fascicles (Kohrs et al., 2011; Hayashi et al., 2013). However, not all tissues are affected the same by aging (Lemmex et al., 2016; Thornton et al., 2015).

Thus, a second challenge for those interested in the regeneration of injured connective tissues is to understand the complexity of the organization and biochemical composition of the tissue that relates to function (Table 1). The composition and organization of the ECM is laid down by a biological response that is coordinated with mechanical stimuli during growth and maturation. An example of this process was shown by experiments depriving knee ligaments of loading in young rabbits which led to a cessation of growth (discussed in Hart et al., 2002; Walsh et al., 1992, 1992). This point has also been reported in other model systems where the Wnt signaling pathway has been implicated in the biological response to mechanical loading (Brunt et al., 2017). In the early post-natal period of humans, mechanical loading of joint tissues, as well as muscles and tendons would lead to tensile loading of muscles, tendons and ligaments, and compression and shear loading of cartilage and menisci via movement, crawling and ultimately walking. However, in the skeletally mature or aged adult there is likely the absence of some of the early anabolic stimuli when an implant may be required, as well as an established biomechanical set point for the integrated tissue of a joint. Likely, some of the anabolic mediators present during growth to maturation which then decline with age include those related to growth hormone and its downstream mediators of the IGF-1 family (Rosenfeld and Hwa, 2009; Yoshida and Delafontaine, 2020; Dixit et al., 2021). Furthermore, during early post-natal life, an individual could also be exposed to growth mediators present in human milk that would be available until lactation ceased (Suwaydi et al., 2021).

### The Involvement of Acute and Chronic Inflammation Following Injury, Surgery and Disease in Connective Tissues

Following an injury to a connective tissue such as the anterior cruciate ligament (ACL), the menisci, or rupture of a tendon, the initial damage to the tissue results in an acute inflammatory response and its consequences. While such an inflammatory response may be limited in duration, it can set off a series of events leading to tissue fibrosis and loss of function. In other circumstances, such as rupture of the ACL, the torn ends of the ligament are separated, and if a reconstruction is not attempted, the ends of the ACL can be resorbed.

Interestingly, if one does reconstruct the ACL with a piece of autologous tendon, or an allograft, or even a tissue-engineered ligament, the surgery itself is an inflammatory stimulus that can impact the success of the operation. Of note, in the circumstance of an ACL reconstruction with a piece of autologous tendon that was initially mechanically stronger than the original ACL, over time the graft begins to exhibit creep, stretch out and lose function. This is likely facilitated by the presence of a more chronic inflammation that leads to the conversion of the tendon graft to scar tissue (discussed in Frank et al., 2004; 2004a).

In other conditions, such as osteoarthritis, the degeneration of the tissues of the joint is accompanied by a chronic inflammation in the intraarticular space (discussed in Woodell-May and Sommerfeld, 2020). Similarly, but more intense, is the intra-articular inflammation associated with rheumatoid arthritis (discussed in Hart et al., 2004). Thus, the environment of a joint exposed to chronic inflammation is very abnormal. Attempts to regenerate damaged or injured tissues in such environments may compromise even the most ideal tissue-engineered replacements unless the environment is altered (discussed in Hart et al., 2004).

Recently, it has been found in preclinical models that treating animals immediately after surgery or an insult to a joint with an anti-inflammatory such as a corticosteroid can diminish the consequences of the inflammation over the longer term (Heard et al., 2015; Heard et al., 2016; Heard et al., 2019; Heard et al., 2021; Barton et al., 2018; Sieker et al., 2016). The efficacy of such interventions has been demonstrated in both porcine models (Sieker et al., 2016) and rabbit models (Heard et al., 2015; Heard et al., 2016; Heard et al., 2019; Heard et al., 2021; Barton et al., 2018). The types of injuries to a joint range from ACL rupture and idealized autologous ACL repair, to a surgical injury mimicked by injection of blood into the joint. Thus, controlling inflammation and creating an environment more conducive to repair is possible and likely is necessary to enhance success.

While the treatments discussed above are likely not optimal as yet, the concept that one can suppress acute inflammation and prevent it from becoming chronic and/or inducing other sequelae such as osteoarthritis-like changes in a joint has been validated and could be translated to patient populations. However, if corticosteroids are used to control inflammation, care must be taken in the dosages used as too high a dose of corticosteroids can have detrimental effects on mesenchymal stem cells (Yasui et al., 2018).

Thus, a third challenge faced by those striving to regenerate injured or damaged connective tissues is to create an environment that will allow an *in vitro* generated tissue-engineered construct to optimally be integrated into the *in vivo* environment and restore function over the long term. (Hart et al., 2004). Not only must a catabolic environment be neutralized, as most need for tissue engineered replacement tissues is in adults one may also have to generate an anabolic environment to enhance the success of the constructs. In other words, the surgery to implant the construct is perceived as an injury by the body, so one has to counteract both the associated inflammation and the consequential infiltration of the graft by scar-like fibrotic tissue and the remodelling that follows (Table 1).

### The Way Forward

When faced with the challenges for success outlined above regarding whether *in vitro* generated tissue-engineered constructs via cells, complex scaffolds and 3D printed composites, as well as other composites containing anabolic molecules such as growth factors are up to the task of regenerating injured or damaged tissues, one needs to perhaps step back and re-evaluate the expectations associated with the term “regeneration”. That is, one needs to define the objective: is there a need for exact biological regeneration (consistent with early mature tissue or tissue at the stage of the remainder of the host?), regeneration of biomechanical integrity, or long-term functional regeneration? With regard to functional regeneration, if an orthopedic surgeon sews the torn ends of a ruptured Achilles tendon back together with formation of scar tissue, the tendon may function quite well for >40 years if the goal is for the patient to be able to return to daily living rather than return to playing soccer or basketball at an elite level. Similarly, an ACL reconstructed with a tissue-engineered construct may be functionally adequate for daily living assuming the inflammation in the joint is controlled early, but the ACL is not fully regenerated. The same can be said perhaps for meniscal replacements. However, for those with osteoarthritis, the goal for regeneration of cartilage and the functionality of the joint as an organ system (Radin et al., 1991, Frank et al., 2004, 2004a; Loesser et al., 2012) may require a tissue engineered construct that is in fact very close to a regenerated cartilage after incorporation. However, if the other tissues of a joint have been influenced by the individual’s life-experiences and epigenetically modified, one may ask if the relationship of the tissue-engineered regenerated tissue to other tissues in the joint will be appropriate (discussed in Frank et al., 2004; 2004a).

Recent studies with regard to regenerating articular cartilage not only provide insights into the boundary conditions associated with regeneration of such cartilage in defects in both preclinical models and more recently, in patients (Shimomura et al., 2018b, 2021), but also opportunities for success. Generation of Tissue-Engineered Constructs (TEC) with mesenchymal stem cells derived from autologous synovium were developed following culture *in vitro* to yield a composite of undifferentiated cells and a matrix generated by the cells themselves without an exogenous scaffold. When implanted into cartilage defects of adult patients (Shimomura et al., 2018b, 2021) or adult or adolescent pigs (Ando et al., 2007; Shimomura et al., 2010), the allogeneic MSC [i.e., male cells into female recipients] in the implanted TEC did not survive in the implant when assessed at 6 months [unpublished observations] but apparently were able to facilitate the migration of endogenous cells into the implanted TEC and their re-establishment of the cellular organization and conversion of the matrix to something more hyaline cartilage-like with a superficial zone, middle zone and deep zone (Fujie et al., 2015). However, after 1 year in the pigs, the resultant cartilage was not perfect in that the lamina splendens did not reform on the surface of the new cartilage (Ando et al., 2012). Thus, even in the adult environment, appropriate biological and mechanical cues are sufficient to evolve a histologically similar and biomechanically capable tissue compared to normal hyaline cartilage. Nevertheless, there is still room for improvement. Of note, the finding that MSC in implanted TEC did not survive is consistent with the literature (de Windt et al., 2017).

It should be noted that use of allogeneic MSC in the preparation of porcine TEC have also been used successfully to repair cartilage defects (Shimomura et al., 2010). Thus, in this circumstance, the MSC do not appear to be immunologically rejected by the host. Because of this apparent immunological privilege, the use of human MSC such as those derived from Wharton’s jelly and other sources have been investigated (for example: Jiang et al., 2021).

In addition, while the TEC approach discussed above has been successful in the treatment of smaller cartilage defects, in many instances, a more extensive injury to meniscus, cartilage or a ligament or tendon may require a different approach, that of using scaffolds to augment implantation into the defects. Thus, incorporation of MSC into scaffolds have been investigated using scaffolds such as decellularized cartilage (Jiang et al., 2021), collagen (Sadlik et al., 2017), silk proteins (Barlian et al., 2020), or scaffolds made of chemical polymers (Liu et al., 2018). Such scaffolds may be biodegradable *in vivo*, but should have mechanical properties that allow for a sufficient time for the seeded cells to respond to the *in vivo* environment.

From some perspectives, articular cartilage may be a relatively simple test case for regeneration as it is avascular and aneural, and while it may be somewhat heterogeneous, it does not have overt transitions in tissue composition and function. Similarly a tendon transitions seamlessly between its enthesis, mid-substance, and myotendinous junction, as well as the innervation and a microvasculature that is restricted to the more surface layers of the tendon (i.e., paratenon or epitenon layers). The organization of a meniscus is more complex than the corresponding articular cartilage with its inner aneural/avascular aspect which sees high compressive loading, a transition zone, and then an innervated/vascular outer zone subjected to large hoop stresses and with two insertion sites into bone (discussed in Andrews et al., 2014; Andrews et al., 2017; Murphy et al., 2019; Markes et al., 2020). The complexity of the matrix with its three-dimensional mosaic array of tie fibres and other collagen and proteoglycan molecules intercalated with cells will be difficult to reproduce *in vitro*. While some of these challenges may be difficult to overcome *in vitro* even with 3D printing of tissue-specific decellularized ECM and cells, it may be that only an approximate replica of the original natural tissue is required. Subsequent further *in vivo* adaptations will occur following implantation as was observed with the TEC for cartilage repair discussed above. In spite of the complexity of the menisci, some successes have been achieved (discussed in Shimomura et al., 2018, 2018a; Mauck and Burdick, 2015).

To achieve the best approximate tissue engineered construct for specific applications will likely require the involvement of a relevant biomechanical stimulus during its preparation. Certainly, applying tensile loading to a tissue engineered ligament construct as it is being developed enhances the mechanical integrity of the resultant tissue (Hart et al., 2005; Goulet et al., 2011, 2014), and potentially, its improved *in vivo* functioning. Depending on the application, the approach of exposing the constructs to mechanical loading may initially require a deformable scaffold or matrix that eventually becomes a composite of molecules secreted by the cells intercalated with the original scaffold as the construct matures (discussed in Hess et al., 2010). Thus, to make sure the cells in a construct are not stress shielded when exposed to a loading protocol *in vitro*, care should be given to the rigidity of any scaffold materials. Supplying biomechanical stimuli to developing tissue-engineered constructs may not only stimulate anabolic responses by the cells, but also inhibit potential catabolic molecules expressed by cells in the absence of mechanical loading (Natsu-ume et al., 2005). Recent developments using the MyoRobot system will allow assessment of the response of tissues such as muscles and cell-biomaterial constructs to defined mechanical loading (Haug et al., 2018; Friedrich et al., 2019). Such approaches will aid in assessing the integration of biology and biomechanics in such constructs.

While the human studies detailing repair of cartilage defects with TEC did not specifically attempt to regulate any post-surgery inflammation, attention perhaps should be directed to ensuring the existence of the best post-surgical environment so that this potential threat to successful regeneration is controlled, as has been shown in the preclinical studies discussed earlier (Heard et al., 2015; Heard et al., 2016; Heard et al., 2019; Heard et al., 2021; Barton et al., 2018; Sieker et al., 2016). Thus, preparing an approximate tissue *in vitro* followed by implantation into the most conducive environment for subsequent success will further enhance the improvement of tissue-engineered constructs, moving the field from tissue repair closer to tissue regeneration and hopefully, long term function. However, it should be noted that attempts to control any inflammation following implantation of TEC in the humans studies (Shimomura et al., 2015; 2018b, 2021) were not implemented so it may depend on the local environment and whether the inflammation is acute or chronic (Hart et al., 2004). This will require further research.

Finally, while not a challenge per se, choosing an appropriate preclinical model is also of critical importance going forward to optimize tissue engineering approaches for regeneration of connective tissues of the MSK system, particularly those of joints such as the knee where the engineered construct would function in a complex mechanical and biological environment. This would be facilitated by having a preclinical model where biology (histology and molecular biology) and biomechanics can be assessed, as well as imaging for longitudinal studies (Hart et al., 2021). Thus, models such as pigs (Ando et al., 2007; Shimomura et al., 2010; Sieker et al., 2016) or sheep (Hart et al., 2021; Brzezinski et al., 2017) should be considered rather than smaller models that are more constrained regarding assessment potential.

## Conclusion

The return of a damaged or injured musculoskeletal tissue to long term function depends on the unique biological requirements of the tissue, the mechanical environment in which it has to function, and the expectations for the use of the tissue. Thus, depending on the circumstances, this may require assessment of where on the continuum of repair to regeneration the situation lies. The goal of regeneration of a tissue that cannot be adequately repaired will require tissue-engineering approaches that have both *in vitro* and *in vivo* challenges to overcome. These include regeneration of a tissue *in vivo* in an environment having some biological and mechanical cues, but lacking many of the anabolic cues present during growth and maturation. Thus, replicating the *in vivo* loading environment during the preparation of a tissue-engineered construct to condition the cells and the construct will likely be beneficial. Finally, there is also a need to control the *in vivo* environment to eliminate adverse conditions such as a catabolically oriented inflammatory state which could interfere with successful implantation of an *in vitro*-optimized construct. Thus, for successful regeneration, one needs to think about what should be introduced to the body to achieve what is needed after incorporation through scar creation and infiltration followed by tissue remodelling. In addition, there needs to be recognition that what is needed may not be a recapitulation of the original as other tissues in the joint will have moved to a new “set point” and may not go back to the original. Achievement of what is needed therefore involves both mechanical and biological compatibility with the other tissues in the joint in their evolved state for sustained functioning thereafter. While some of these challenges may be daunting, they likely can be overcome using innovative approaches. Some of these are summarized in Table 1.
